# "Evaluating the model of classification and valuation of disabilities used in Brazil and defining the elaboration and adoption of a unique model for all the country": Brazilian Interministerial Workgroup Task

**DOI:** 10.1186/1471-2458-11-S4-S10

**Published:** 2011-05-31

**Authors:** Heloisa Di Nubila, Ana Rita de Paula, Miguel Abud Marcelino, Izabel Maior

**Affiliations:** 1 Faculty of Public Health – University of São Paulo, São Paulo, Brazil; 2 NGO – CVI Araci Nallin (Centro de Vida Independente – ILC Independent Life Center), São Paulo, Brazil; 3 National Social Security Institute, Medical Experts Division, Petrópolis-RJ, Brazil; 4 Sub-Secretary for the Rights of Persons with Disability – Brazilian Special Presidential Secretary of Human Rights, Brasília, Brazil

## Abstract

The President of Brazil established an Interministerial Work Group in order to “evaluate the model of classification and valuation of disabilities used in Brazil and to define the elaboration and adoption of a unique model for all the country”. Eight Ministries and/or Secretaries participated in the discussion over a period of 10 months, concluding that a proposed model should be based on the United Nations Convention on the Rights of Person with Disabilities, the International Classification of Functioning, Disability and Health, and the ‘support theory’, and organizing a list of recommendations and necessary actions for a Classification, Evaluation and Certification Network with national coverage.

## Introduction

According to 2000 Brazilian Census [1], there are 24.5 millions of persons with any type of functional limitation, which corresponds to 14.5% of the population, with 70% living under the line of poverty.

There is a comprehensive legal framework for the promotion and protection of persons with disabilities rights that has its origin in the Brazilian Federal Constitution [Table 1 – Item A]. The National Policy for Integration of Persons with Disabilities [Table 1 – Item B] and the sectorial policies are accomplished through affirmative actions and equalization of benefits, as an effort to mitigate the existent social problems, by reason of a historical process, and to promote inclusion.

**Table 1:** Disability in Brazil: legislation benchmarks

Item	Law/Date	Subject /Address
A	Constitution of the Federative Republic of Brazil October 30^th^ 1988	http://www.planalto.gov.br/ccivil_03/constituicao/constitui%C3%A7ao.htm

B	Law 7853 October 24^th^ 1989	Disposes on National Policy for the Integration of Persons with Disabilities http://www.planalto.gov.br/ccivil_03/Leis/L7853.htm

C	Decree 3298 December 20^th^ 1999	Regulates Law 7853 of 24/10/1989, provides for the National Policy for the Integration of Persons with Disabilities, consolidates the rules for protection, and other matters http://www.planalto.gov.br/ccivil/decreto/d3298.htm

D	Decree 5296 December 2^nd^ 2004	Regulates Law 10048 of 8/11/2000, which gives priority to support people specified, and Law 10098 of 19/12/2000, laying down general standards and criteria for the promotion of accessibility http://www.planalto.gov.br/ccivil/_ato2004-2006/2004/decreto/d5296.htm

E	Decree 0-003 September 26^th^ 2007	Establishes an Interministerial WorkGroup in order to evaluate the model of classification and valuation of disability used in Brazil and set the elaboration and adoption of a single model for the whole country http://www.planalto.gov.br/ccivil_03/_Ato20072010/2007/Dnn/Dnn11354.htm

F	Decree 186 July 9^th^ 2008	Approves the text of the Convention on the Rights of Persons with Disabilities and its Optional Protocol, signed in New York on March 30, 2007 http://portal.mj.gov.br/corde/arquivos/doc/DECRETO%20LEGISLATIVO%20N.doc

G	Decree 6214 September 26^th^ 2007	Regulates the Benefit of Continuous Providing (BPC) of social assistance due to the person with disabilities and the elderly according to the Law 8742 of 7/12/1993 and Law 10741 of 1/10/2003, adding a paragraph to art. 162 of Decree 1048 of 6/5/1999, and other matters OuvirLer foneticamente http://www.planalto.gov.br/ccivil_03/_ato2007-2010/2007/decreto/d6214.htm

Currently, many fragmented instruments are being used for the categorization of disabilities, based on the definitions expressed in Decree 3298/1999 [Table 1 – Item C] and its modifications in Decree 5296/2004 [Table 1 – Item D]. Since these definitions are centered on diseases and corporal structure alterations, they reflect a purely medical model, which could be represented by the International Classification of Diseases (ICD-10) [2], without considering the functioning of the person or a valuation system.

Recognizing who are the persons with disability and which of them need the different social protection policies is one of the duties of the Government to promote equity in opportunities and to warrant human rights and fundamental freedom.

Through a Decree (Sept 26th 2007) [Table 1 – Item E], the President of Brazil established an Interministerial Work Group (IMWG) in order to “evaluate the model of classification and valuation of disabilities used in Brazil and to define the elaboration and adoption of a unique model for all the country”.

The IMWG searched for models and tools used in other countries in order to submit a proposal consistent with the new concept defined by the UN Convention on the Rights of Persons with Disabilities [3], ratified by the Legislative Decree 186/2008 [Table 1 – Item F] with the equivalence of a constitutional act.

The IMWG worked for a period of 6-10 months: nine ordinary meetings were organized to discuss the theme, from June to December 2008, and an extraordinary meeting was held in May 2009; in addition, there were discussion subgroups, IMWG members electronic communications, and specialists from different Associations were listened to. The IMWG worked on:

• presentations of the current definitions of disability, based on the different kinds of benefits and consequently linked to different Ministries;

• the search for international publications about laws, problems with definitions, and parameters used for defining eligibility for social benefits all over the world;

• the elaboration of a proposal.

The Ministries involved in the IMWG were the following: 1) Human Rights Special Presidential Secretary – National Coordination of Integration of Persons with Disabilities (CORDE); 2) Ministry of Health– Coordination of Health for Persons with Disabilities; 3) Ministry of Social Security – National Institute of Social Security (INSS); 4) Ministry of Social Development and Actions against Hunger – Department for Continuous Benefit for the Aged and Persons with Disabilities; 5) Ministry of Work; 6) Ministry of Education; 7) Ministry of Transport; and 8) Planning Ministry. Specialists from Universities and representatives of NGOs of Persons with Disabilities were also invited.

## Definitions of disability

The definition of the condition of a person with a disability is linked to a historical process, supported by theoretical and philosophical principles.

In the UN Convention preamble [3], it is stated that disability is a concept in evolution and that it is the result of the interaction between persons and barriers due to attitudes and environments that impede the full and effective participation of these persons in the society with equal opportunities. Article 1 of the UN Convention [3] enunciates that “persons with disabilities include those who have long-term physical, mental, intellectual, or sensory impairments which in interaction with various barriers may hinder their full and effective participation in society on an equal basis with others”.

Such concepts grounded the technical conclusions of the IMWG, which recognized disability as a phenomenon situated in the biological, psychic, social and political interface of the individual. In the bio-psychosocial model of the International Classification of Functioning, Disability and Health (ICF) [4], disability is defined as more comprehensive than impairment, being a generic term that includes impairments, activities limitation, and participation restriction. The term disability indicates principally the negative aspects of the interaction between an individual (with a health condition) and the contextual factors (environmental and personal factors), involving a dynamic relation.

An individual could present an impairment (body level) and not necessarily experience a disability. On the other hand, a person could experience a disability without having any impairment, only by reason of stigma or prejudice (attitude barrier). As a health classification, ICF could, by its coverage and extension, contribute to achieve coherence issues and, through its qualifiers, relevance issues for the definition of disability [5].

### Definitions of disability used in Brazil

In the Brazilian public policies, several definition criteria, instruments, and methods co-exist for the characterization of disability that originate from distinct conceptions constructed in different historical periods. Most of these criteria, instruments and methods are incompatible with the definition of the UN Convention [3].

The instruments and methods used in other countries, some of which in conformity with the definition of the UN Convention [3], do not apply integrally to the Brazilian reality, given the interdependence of the characterization of disability with the socio-cultural situation.

The diversity of methods generates distortions in the application of policies, as well as fragmentation of services and, consequently, a waste for the Brazilian citizen with a disability, who is obliged to seek different public organs and documents for accessing his/her own rights.

It is necessary to create a unique model of classification and valuation, which will originate tools adequate to the public policies for Brazilian citizens with disabilities, coherent with the following directives:

1 it will not be based uniquely on clinical diagnosis of diseases, conditions or traumatic lesions;

2 it will not be based uniquely in sequels diagnosis (organic consequences) of diseases, conditions or traumatic lesions (ex: monoparesis, paraplegia, aphasia, low vision); and

3 it will consider, simultaneously and equanimously, personal factors (gender, instruction level, age, coping/resilience), environmental factors (accessibility, supports, attitudes), and social and economic factors that could favor or hamper the performance in activities and participation (functioning), as well as body structures and functions.

The proposed model shall to be in harmony with the ICF model [4], emphasizing that ICF is a classification, not an evaluation tool, since it does not establish the limits of who is or who is not a person with a disability.

The valuation should consider the nature, the type, and the frequency of supports that the person with a disability needs, according to the ‘support theory’ (SIS, Supports Intensity Scale – AAIDD, formerly AAMR) [6]. The concepts presented in the SIS are absolutely compatible with the concept of facilitators in ICF.

Independently of disease or organic sequel (monocular vision, palatal cleft, albinism, chronic disease, etc.) any person could be evaluated by the proposed model and have the disability valuated, according to important alterations in his/her functioning and considering the influences of social, economic, and environmental factors, among others. In this way, not any disease would be excluded, but the valuation of impairment or disability would depend on the global evaluation of the individual, based on ICF and the ‘support theory’.

As the disability concept is dynamic, the normatization that defines the model of classification and the respective tools shall permit changes that will follow the evolution of the inner concept.

## Directives to implement a new model of disability

Currently, each organ of public authority, responsible for the implementation of sectorial policies and affirmative actions, responds to the demands and necessities of the persons with disability in an unarticulated manner, creating different mechanisms, practices and services for verification of the disability condition.

The legislative power, sensible to the particular demands of specific population groups, in the absence of a unique model guiding its practices, tends to propose an addition of categories based exclusively on diseases and sequels, with an increase in the attended population and without necessarily benefits for the most socially vulnerable and most destitute citizens with a disability, i.e., without guarantee of fairness or equity.

The use of financial resources for benefits and other special actions could be optimized if the evaluations were not so fragmented and if there were more coherence among the instruments. Up to now, there is not enough knowledge about the social or financial impact of the application of resources and its effect on the quality of life of persons contemplated or not by the benefits and affirmative actions.

The evaluation of disability should originate a unique certification that would give a more equitable access to several affirmative actions or benefits. This unique certification makes it necessary the creation of an evaluation and certification network with national coverage.

The certification would be issued based on the evaluation performed by multiprofessional teams, with an interdisciplinary proceeding model, specifying the benefit and/or affirmative actions for which the person with a disability is eligible. These teams should be trained for the acquisition of the skills and specific knowledge necessary for the implementation of the model and the application of the evaluation instruments.

In order to define the model details to elaborate instruments and routines, as well as to validate these instruments, it will be necessary to perform a short- to medium-term study, preferentially done by a research institution that presents the technical conditions for its execution.

Monitoring and evaluation of the results and impact, social and economic, of the implementation of the model shall also be done by a research institution with the adequate technical conditions.

## Conclusions and recommendations

The group proposes the implementation of the UN Convention [3] concept for the construction of the conceptual model of disability to be adopted and for the development of instruments of classification, valuation and certification.

### Short term recommendations

#### Model details

The proposed model shall respond to the complexity of the disability condition, articulating the social, economic, organic and contextual factors (personal and environmental) that could favor or hamper the performance in activities and participation (functioning) of a person with disability, not giving “a priori” differentiated weights to these factors, treating them in a balanced manner during the process of the subject particularization.

The model shall be comprehensive in order to permit adjustment to the diverse natures of policies, programs and actions for fighting against discrimination and social marginalization and for implementing affirmative measures through the concession of benefits and/or services, as well as to permit the construction of differentiated evaluation instruments, according to eligibility criteria of the target population, depending on the objective of each benefit. This means that there will be persons with disability that will be eligible for some benefits and not for others.

It is important to use previous experiences, such as that of Benefit of Continuous Providing - BCP (Benefício de Prestação Continuada /BPC in Portuguese) [Table 1 – Item G], for the construction of instruments.

Considering all the previous arguments, it is necessary to detail the model through studies of applicability in the different real situations.

#### Instruments, routines and practices

It is necessary to create national instruments, routines and practices that reflect the model and these shall be:

- anchored in the concept of disability of the UN Convention in philosophical, theoretical and conceptual terms, as well as in the ICF and in the ‘support theory’;

- specific and adequate to each finality of benefits and affirmative measures;

- viable and appropriate for the diversity of Brazilian regional realities, considering for example the infra-structure of the resources network;

- responsive to the nature of existing benefits and affirmative actions;

- able to incorporate the evolution of policies.

As these instruments, routines and practices are innovative, without historical accumulation of experiences, it is necessary to proceed with a process of validation, including presentation for public consultation.

#### Classification, valuation and certification network

In the absence of a network that makes possible the implementation of this policy, a synergy of efforts in the health, social assistance and social security areas is necessary, optimizing the existent resources for the implementation of a coordinated network with national coverage, decentralized and regionalized, for the classification, valuation and certification of disabilities, according to the following steps:

- determination of directives for the network implementation by the ministries involved, concordant with the before mentioned directives;

- inventory of existing material and human resources;

- elaboration of contents, methods and infra-structures for the development of a program of orientation of the teams that will act in the network;

- definition of the subsequent phases with medium term goals.

### Medium term recommendations

For the implementation of the Classification, Valuation and Certification Network, it is necessary to continue to work with the goals defined in the previous phase, understanding the complexity and the dimension of the task. As this network is an innovative proposal, involving an interministerial management of services, it is necessary to develop monitoring of its operation and of the results achieved.

It is also very important to evaluate the results and social-economic impact of any public policy, especially if this policy involves the objective of equalization of opportunities and reparation of historical situations of discrimination and social marginalization, as is the case of concession of assistance and social security benefits and of affirmative measures, including the actions for the guarantee of the rights of persons with a disability.

To understand the repercussions on the normative acts of public management, it is desirable to study the existent laws and decrees, observing the coherence between the UN Convention and the proposed model.

The adoption of a unique model for the classification and valuation of disability will require an assessment of the repercussions on the public budget and a cost-benefit analysis. It will be also important to study the necessity of proposing other normative acts in order to ensure the adoption and implantation of the proposed model.

## Expected products

It is expected that this process will result in a technical document containing the justified details of the Brazilian model proposed, as well as instruments of classification, valuation and certification of disability, according to the different benefits and affirmative measures existent in the Brazilian legal framework, and recommendations for the implementation of the Network for Classification, Valuation and Certification (including aspects regarding management, operation, human resources, organogram and flow chart, as well as goals, methods, costs and systems involved in its operationalization). Another technical document shall be produced containing: 1) suggestion of indicators and instruments for the follow-up/monitoring of the model and its instrumental application, as well as of the Classification, Valuation and Certification Network performance; 2) suggestion of indicators and instruments for evaluating the results and the social/economic impact of the adoption of the proposed model; 3) estimation of costs and impact on the public budget with the adoption of the Brazilian model; and 4) analysis of the cost-benefit relation of the concession of benefits of assistance and social security, as well as of affirmative measures existent in the Brazilian legal framework.

## List of abbreviations used

AAIDD: American Association of Intellectual and Developmental Disabilities

AAMR: American Association of Mental Retardation

BCP: Benefit of Continuous Providing (in Portuguese- BPC – Benefício de Prestação Continuada)

IMWG: Interministerial Workgroup; ICF: International Classification of Functioning Disability and Health

SIS: Supports Intensity Scale.

## Competing interests

The authors declare that they have no competing interests.

## References

[B1] Instituto Brasileiro de Geografia e Estatística (IBGE) Censo 2000 http://www.ibge.gov.br/home/presidencia/noticias/20122002censo.shtm

[B2] World Health Organization ICD-10. International Statistical Classification of Diseases and Related Health Problems, Tenth Revision 2004 Geneva: World Health Organization

[B3] United Nations Draft Convention on the Rights of Persons with Disabilities http://www.un.org/esa/socdev/enable/rights/ahc8adart.htm#art1 10.1515/9783110208856.203 18348362

[B4] World Health Organization ICF. International Classification of Functioning, Disability and Health 2001 Geneva: World Health Organization 19537677

[B5] Di Nubila HBV Aplicação das classificações CID-10 e CIF nas definições de deficiência e incapacidade/ Application of the classifications ICD-10 and ICF on definitions of disability Doctoral thesis 2007 Faculdade de Saúde Pública da Universidade de São Paulo

[B6] Thompson JR Bryant BR Campbell EM Craig EM Hughes CM Rotholz DA Schalock RL Silverman WP Tassè MJ Wehmeyer ML Supports Intensity Scale – User’s Manual 2004 Washington: American Association of Mental Retardation (AAMR)

